# Dose-response relationship of ICS/fast-onset LABA as reliever therapy in asthma

**DOI:** 10.1186/s12890-019-1014-4

**Published:** 2019-12-28

**Authors:** Richard Beasley, James Harper, Grace Bird, Harriette Dunphy, Alex Semprini, Ian D. Pavord, Alberto Papi, Mark Weatherall

**Affiliations:** 1 Medical Research Institute of New Zealand, Private Bag 7902, Wellington, New Zealand; 2 Victoria University of Wellington, PO Box 600, Wellington, New Zealand; 3 Capital & Coast District Health Board, Private Bag 7902, Wellington, New Zealand; 4 Oxford Respiratory NIHR BRC, Nuffield Department of Medicine, University of Oxford, Old Road Campus, Roosevelt Drive, Oxford, OX3 7FZ UK; 5 University of Ferrara, University Hospital S.Anna, 44121 Ferrara, Italy; 6 University of Otago Wellington, PO Box 7343, Wellington, New Zealand

**Keywords:** Asthma, Dose-response relationships, Inhaled corticosteroids, Long acting beta agonists, Severe exacerbations

## Abstract

**Background and objective:**

The dose-response relationship of inhaled corticosteroid (ICS)/fast-onset long acting beta agonist (LABA) reliever therapy has not been formally addressed. The objective of this retrospective analysis is to ascertain from the available evidence whether ICS/fast-onset LABA administered as reliever therapy has a different dose-response relationship than maintenance fixed dose ICS/fast-onset LABA therapy in reducing risk of severe exacerbations.

**Methods:**

A systematic literature review was undertaken to identify randomised controlled trials (RCTs) in which randomised treatments included either i) budesonide/formoterol reliever monotherapy versus budesonide/formoterol fixed dose maintenance with short acting beta agonist (SABA) reliever therapy, or ii) budesonide/formoterol reliever therapy in addition to budesonide/formoterol maintenance versus higher fixed dose maintenance budesonide/formoterol with SABA as reliever therapy. Eligible studies were reviewed to allow determination of the relative potency and efficacy of the comparator regimens to reduce the risk of a severe exacerbation.

**Results:**

The one RCT of budesonide/formoterol reliever monotherapy showed a 4.6-fold (95% CI 2.9 to 7.3) greater potency than budesonide/formoterol fixed dose maintenance plus SABA reliever therapy in reducing the risk of severe exacerbations. In the one RCT that compared budesonide/formoterol maintenance and reliever therapy with higher fixed dose maintenance budesonide/formoterol plus SABA reliever therapy, there was an additional 26% (95% CI 4 to 42%) reduction in severe exacerbation risk with the addition of budesonide/formoterol reliever therapy to maintenance budesonide/formoterol, despite a 25% lower total budesonide/formoterol dose.

**Conclusion:**

The limited available evidence suggests that budesonide/formoterol reliever therapy has greater potency and efficacy than budesonide/formoterol fixed dose maintenance plus SABA reliever therapy in reducing the risk of a severe exacerbation. This is an important concept which has the potential to guide clinical practice in asthma, although the small number of studies available highlights the need for further research to better define these pharmacological properties.

## Background

In adolescent and adult asthma, inhaled corticosteroid (ICS)/long acting beta agonist (LABA) therapy has shown efficacy when prescribed according to three regimens: as a fixed maintenance dose ICS/LABA together with a short-acting beta agonist (SABA) for relief, as an ICS/fast-onset LABA for both maintenance and reliever therapy, or as an ICS/fast-onset LABA as sole reliever therapy [1–6]. Clinicians need to be familiar with the comparative dose-response relationships of these regimens to enable their optimal implementation in clinical practice. However, the comparative dose-response relationships of these ICS/LABA regimens have not been formally assessed.

The aim of this manuscript is to evaluate evidence from published randomised controlled trials (RCTs), to compare the dose-response relationships of combination ICS/fast-onset LABA when used either as reliever therapy or regular maintenance therapy. The key question was whether there is evidence that ICS/fast-onset LABA administered as reliever therapy has a different dose-response relationship compared to administration as fixed dose ICS/fast-onset LABA maintenance therapy in reducing severe exacerbations. An initial systematic review identified that budesonide/formoterol was the only combination ICS/fast-onset LABA product studied in RCTs that were potentially eligible for inclusion in the proposed analysis. As a result, for the purposes of testing this hypothesis we limited our analysis to data from RCTs of budesonide/formoterol as the relevant ICS/fast-onset LABA.

## Methods

A systematic search of PubMed, supplemented by a hand search of respiratory journals, was used to identify all randomised clinical trials that investigated budesonide/formoterol reliever therapy. We searched for the following terms: (“single inhaler” or SiT or SMART or MART or combin* or “maintenance and reliever therapy” or “reliever therapy”) AND (formoterol or eformoterol) AND (budesonide).

A total of 546 studies were identified using the above search terms. Results were filtered to include human studies written in English language. Additional data was requested from the corresponding authors of studies where data for our nominated primary and secondary outcomes were not reported in the primary paper.

### Inclusion and exclusion criteria

Two people examined each paper’s title and abstract, followed by review of the full paper as necessary. In order to be included in the analysis, RCTs had to meet all of the following inclusion criteria:

i. Randomised, controlled clinical trial
ii. Adults and/or adolescents with asthma
iii. Report data on measures of efficacy, including severe exacerbations
iv. Budesonide/formoterol was administered according to one of the two following study design categories:

1. To enable assessment of potency, that there were two randomised arms comprising budesonide/formoterol reliever monotherapy and budesonide/formoterol fixed dose maintenance with a SABA as reliever therapy.
2. To enable assessment of efficacy, that there were two randomised arms comprising budesonide/formoterol reliever therapy in addition to budesonide/formoterol maintenance, and higher fixed dose maintenance budesonide/formoterol with a SABA as reliever therapy. For this assessment the maintenance budesonide dose for the treatment arm of higher fixed dose maintenance therapy needed to be at the level that achieves the maximum achievable benefit, at least 640 μg budesonide (delivered dose) per day [7].

Studies were excluded if any of the following criteria were met:

i. Budesonide/formoterol was administered in more than one device and/or from more than one inhaler.
ii. Within a randomised treatment group, more than one dose of budesonide/formoterol was used as fixed dose maintenance therapy and/or more than one dose of budesonide/formoterol was used as the maintenance element of maintenance and reliever therapy.

### Data extraction

Extraction of data was based on reported data summaries. These included counts and proportions of the number of participants in each treatment arm with a categorical outcome of interest. For continuous variables, we extracted means and standard deviations and the number of participants with these outcomes. The standard deviations, when not explicitly reported, were derived from reported standard errors or confidence intervals. The primary efficacy outcome variable for this analysis was risk of severe exacerbations, defined as the reported number of participants with at least one exacerbation, divided by the number of participants randomised to the treatment regimen, and with severe exacerbations defined according to the American Thoracic Society (ATS)/European Respiratory Society (ERS) criteria [8]. Secondary outcome variables were a measure of asthma control (preferably the Asthma Control Questionnaire (ACQ) score), a measure of lung function (preferably the forced expiratory volume in one second (FEV_1_) and budesonide/formoterol doses.

### Data analysis

Two eligible studies were identified: one in the first design category, for assessment of potency; and one in the second design category, for assessment of efficacy. Assessment of the risk of bias was undertaken according to standard recommendations [9].

The assessment of potency was evaluated in two stages. The first stage was to estimate the relative risk of a severe exacerbation based on the counts of participants with at least one severe exacerbation in each treatment arm, together with its confidence interval. The second stage was to then evaluate the relative potency, in relation to the mean total cumulative dose of budesonide in each randomised arm. In the second stage, under the simplifying assumption that the ratio of mean doses of budesonide in the budesonide/formoterol combination therapy in each of the two treatment arms has a one-to-one relationship with the relative risk of severe exacerbation, dividing the relative risk of exacerbation and its confidence interval by this ratio should approximate a confidence interval for potency. This is similar to the pharmacological principle that relative potency can be estimated by the ratio of doses required to achieve the same therapeutic effect [10, 11].

The assessment of efficacy was based on the comparison between budesonide/formoterol reliever therapy combined with budesonide/formoterol maintenance therapy, versus higher fixed dose budesonide/formoterol maintenance therapy (at the top of the known dose-response curve for the ICS component) combined with SABA as reliever therapy. The difference in efficacy was expressed as the relative reduction in the risk of severe exacerbations between the two regimens. This is similar to the pharmacological principle that relative efficacy can be estimated from the difference in the maximum obtainable effect achieved by doses at or beyond the top of the therapeutic dose response curve [10, 11].

SAS version 9.4 was used.

## Results

**Fig. 1:**
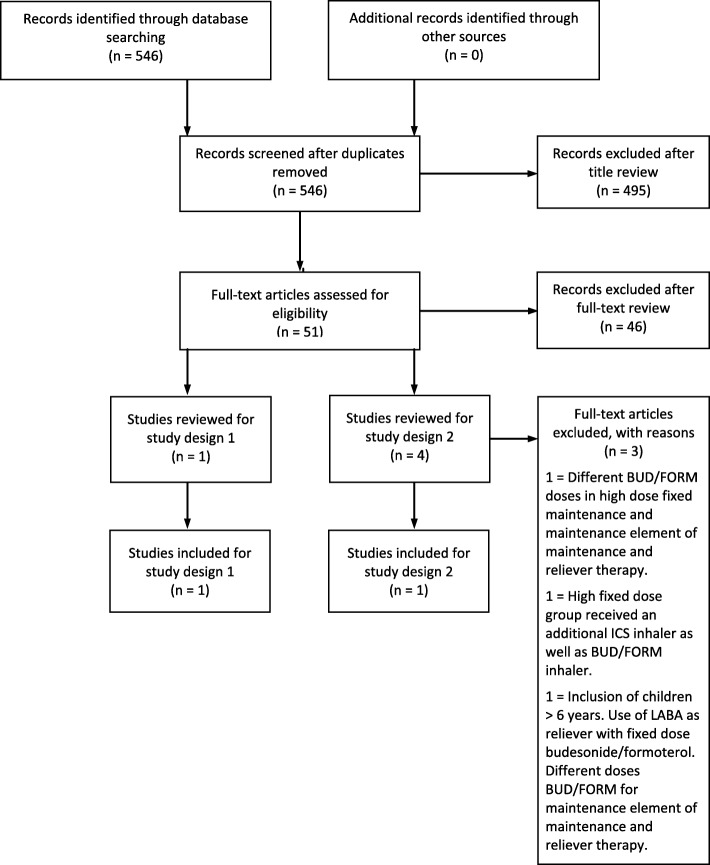
The process of inclusion of studies in the systematic review. Abbreviations: BUD: Budesonide. FORM: Formoterol

There was one study [12] identified that met the criteria for study design 1. Four studies [13–16] were identified that potentially met the criteria for study design 2 [Fig. 1].

Three of these studies were found to be ineligible based upon the inclusion and exclusion criteria described above [14–16]. Thus there was one study that met the inclusion criteria for study design 2 [13].

**Fig. 2:**
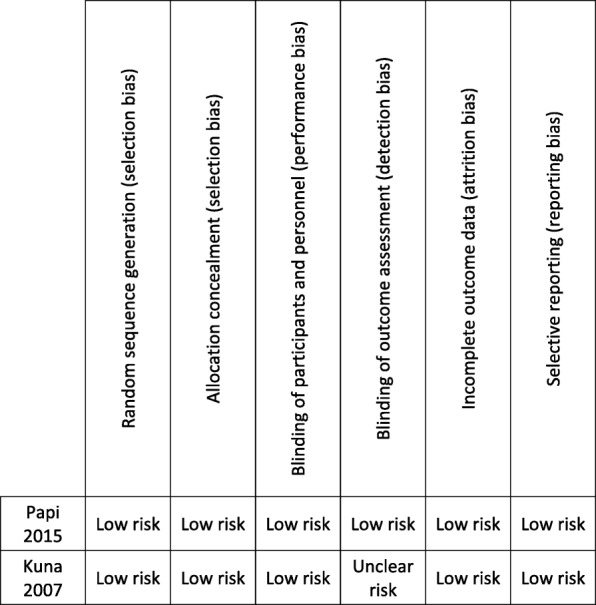
Assessment of bias

There was low risk of bias in these two studies which were included [Fig. 2].

### Study design 1: potency evaluation - budesonide/formoterol reliever monotherapy versus budesonide/formoterol fixed dose maintenance therapy

A single study with this design was identified [12]. In this study, participants with moderate persistent asthma whose symptoms were either not controlled by low-dose ICS (≤500 μg beclomethasone per day or equivalent) or controlled by a fixed combination of low-dose ICS and LABA twice daily in the 2 months before the study were randomised to either budesonide/formoterol 160 μg/4.5 μg (delivered dose; 200 μg/6 μg metered dose), as required for relief of symptoms, or twice daily budesonide/formoterol 160 μg/4.5 μg, with terbutaline as required for relief of symptoms. The proportion of participants with at least one severe exacerbation during the study was 31/424 (7.3%) in the budesonide/formoterol reliever monotherapy group and 31/442 (7.0%) in the budesonide/formoterol maintenance group [Table 1].

**Table 1:** Study Design 1. Budesonide/formoterol reliever therapy vs budesonide/formoterol fixed dose maintenance therapy

Papi et al.^12^	Reliever Therapy	Maintenance Therapy	Relative Risk (95% CI)	*P*
Risk of severe exacerbations:				
(No. participants with at least one severe exacerbation/ No. participants randomised to treatment regimen)	31/424 (7.3%)	31/442 (7.0%)	1.04 (0.65 to 1.68)	0.87
			Ratio	
Cumulative dose:				
Budesonide (mg)	24.5	116.8	0.21	
Formoterol (mg)	0.69	3.2	0.22	
Potency:			4.6 (2.9 to 7.3)	
			Difference (95% CI)	
FEV_1_ (L, change from baseline, mean (SD))	−0.16 (0.37)	−0.01 (0.34)	0.15 (0.09 to 0.20)	< 0.0001
ACQ score (mean change from baseline)	0.25 (0.92)	0.06 (0.74)	−0.21 (− 0.34 to − 0.08)	< 0.002

Abbreviations: *FEV*_1_: Forced expiratory volume in one second; *ACQ*: Asthma Control Questionnaire

The estimated relative risk (95% CI); reliever versus maintenance was 1.04 (0.65 to 1.68), *P* = 0.87. The reported mean cumulative dose of budesonide/formoterol was 24.5/0.69 mg in the reliever group and 116.8/3.2 mg in the maintenance group. Dividing the point estimate and confidence interval for the relative risk by this ratio of ICS/LABA doses gives an approximate point estimate and confidence interval for potency for reliever compared to maintenance ICS of 4.6 (2.9 to 7.3) This is consistent with budesonide/formoterol being approximately three to seven times more potent in reducing the risk of severe exacerbations, when used as reliever monotherapy compared with regular maintenance use.

In contrast, the secondary clinical outcomes of ACQ score and FEV_1_ were significantly improved with maintenance budesonide/formoterol therapy. As a result relative potencies for these clinical outcome measures could not be calculated.

### Study design 2: efficacy evaluation-budesonide/formoterol maintenance and reliever therapy versus higher fixed dose budesonide/formoterol maintenance therapy

One study with this design was included [13]. In this study, participants with asthma and bronchodilator reversibility with FEV_1_ ≥ 50% predicated who had been using ICS for ≥3 months and who had ≥1 exacerbation in the last 1–12 months were randomised to one of three arms: budesonide/formoterol 160/4.5 μg (delivered dose; 200/6 μg metered dose) one inhalation twice daily maintenance therapy plus budesonide/formoterol 160/4.5 μg as required for relief of symptoms; budesonide/formoterol 320/9 μg (delivered dose; 400/12 μg metered dose) one inhalation twice daily maintenance therapy with terbutaline as required for relief of symptoms; or fluticasone/salmeterol 125/25 μg (metered dose) two inhalations twice daily maintenance therapy (equivalent to 640 μg budesonide and 18 μg formoterol per day delivered dose), plus terbutaline as required for relief of symptoms.

The budesonide/formoterol maintenance and reliever group had a decreased risk of severe exacerbations compared with the higher fixed dose budesonide/formoterol group, with the proportion of participants with at least one severe exacerbation 94/1107 (8.5%) and 126/1105 (11.4%) respectively, a relative risk (95% CI) of 0.74 (0.58 to 0.96), *P* = 0.02 [Table 2].

**Table 2:** Study Design 2 - Budesonide/formoterol maintenance and reliever therapy vs higher fixed dose budesonide/formoterol maintenance therapy

Kuna et al.^13^	Maintenance and Reliever Therapy	Higher Fixed Dose Maintenance Therapy	Relative Risk (95% CI)	*P*
Risk of severe exacerbations:				
(No. participants with at least one severe exacerbation/ No. participants randomised to treatment regimen)	94/1107 (8.5%)	126/1105 (11.4%)	0.74 (0.58 to 0.96)	0.02
Mean dose:			Ratio	
Budesonide (μg/day)	483 (320 maintenance; 163 reliever)	640	0.75	
Formoterol (μg/day)	13.6 (9 maintenance; 4.6 reliever)	18	0.75	
			Difference (95% CI)	
FEV_1_ (L, mean (SD))	2.69	2.66	0.01 (−0.03 to 0.04)	–
Asthma symptoms score (mean total score)	1.06	1.07	0.00 (−0.07 to 0.06)	–

Abbreviations: *FEV*_1_: Forced expiratory volume in one second

The calculated dose ratio for ICS/LABA administration was 0.75; derived from the mean daily dose of budesonide/formoterol of 483/13.6 μg for budesonide/formoterol maintenance (320/9 μg) and reliever (163/4.6 μg) therapy, and 640/18μg for the higher maintenance budesonide/formoterol regimen. As a result, when added to budesonide/formoterol 320/9 μg/day maintenance therapy, budesonide/formoterol 163/4.6 μg/day as reliever therapy had greater efficacy than an additional budesonide/formoterol 320/9 μg/day as maintenance therapy.

In contrast, the ACQ and FEV_1_ were similar between the two regimens, suggesting similar efficacy for these clinical outcomes.

### Schematic dose response curves

**Fig. 3:**
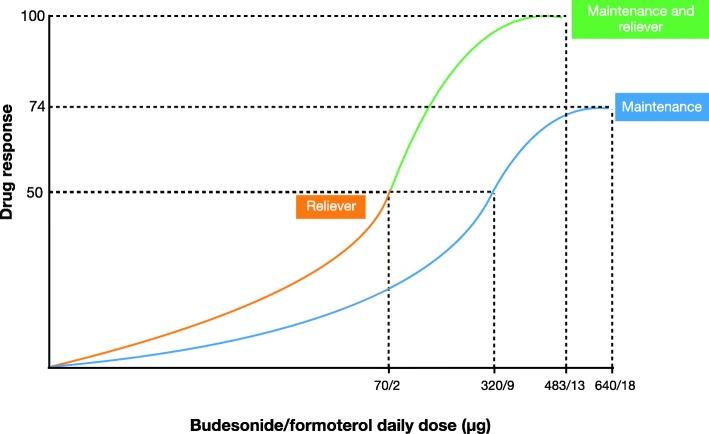
Schematic dose-response curves of severe exacerbation risk for the budesonide/formoterol reliever therapy (orange/green) and the maintenance budesonide/formoterol therapy (blue) regimens based on data presented in this review. The X-axis represents the daily dose of budesonide/formoterol on a logarithmic scale. The Y-axis represents the response in terms of reduction in risk of a severe exacerbation. There were a number of assumptions made in deriving this figure: i) the budesonide/formoterol reliever therapy curve is derived from the study of budesonide/formoterol reliever monotherapy [12] (orange) and the study of budesonide/formoterol reliever therapy used in addition to maintenance budesonide/formoterol therapy [13] (green). ii) the therapeutic effect of budesonide/formoterol reliever monotherapy is set as 50% of the maximum drug response and the therapeutic effect of budesonide/formoterol maintenance and reliever therapy is set as 100% of the maximum drug response. iii) the shape of the log exponential dose-response curve is assumed

The schematic dose-response curves of severe exacerbation risk for budesonide/formoterol reliever therapy and maintenance budesonide/formoterol therapy regimens are shown in Fig. 3.

## Discussion

In this retrospective analysis, we have identified from the limited available evidence that budesonide/formoterol reliever therapy has greater potency and efficacy than budesonide/formoterol fixed dose maintenance plus SABA reliever therapy in reducing the risk of severe exacerbations. The difference for potency was substantial with an estimated 4.6 (2.9 to 7.3) fold difference with budesonide/formoterol reliever monotherapy compared with budesonide/formoterol fixed maintenance therapy. There was an additional 26% (4 to 42%) reduction in severe exacerbation risk with budesonide/formoterol reliever therapy when added to maintenance budesonide/formoterol compared with higher fixed dose maintenance budesonide/formoterol therapy and SABA reliever therapy, despite a 25% reduction in total budesonide/formoterol dose. For the secondary clinical outcomes, there were no substantial differences in efficacy between the two regimens, and although it was not possible to calculate differences in potency, fixed dose maintenance therapy was associated with a statistically but not clinically significant improvement in FEV1 and ACQ score compared to budesonide/formoterol reliever therapy.

There are a number of methodological issues crucial to the interpretation of these findings. Firstly, despite the extensive literature search, the only RCTs eligible for inclusion used budesonide/formoterol as the ICS/fast-onset LABA. Consequently, the generalisability of the findings of this study beyond budesonide/formoterol to other ICS/formoterol products is limited to some extent. There were only two RCTs eligible for inclusion in this study which reduced the confidence in the estimates of the differences observed, however, they were large, well powered RCTs with low risk of bias [12, 13].

Secondly, there is a therapeutic contribution of ICS and LABA components of both the reliever and maintenance therapy regimens. Therefore, the finding of increased potency and efficacy of budesonide/formoterol when used as a reliever compared to a maintenance regimen must be viewed in the context of this combination. Previously, budesonide in combination with formoterol as reliever therapy has been shown to reduce risk of severe exacerbations by 33% when compared with formoterol reliever therapy alone [17]. Furthermore, the addition of formoterol to budesonide as fixed maintenance therapy has been associated with a 17% reduction in severe exacerbation risk [18].

When comparing the efficacy of budesonide/formoterol maintenance and reliever therapy and higher fixed dose budesonide/formoterol maintenance and SABA reliever therapy, the effect of the additional SABA reliever use cannot be separated from the overall efficacy of this latter regimen. If SABA reliever therapy has a beneficial clinical effect, then use of SABA will lead to an underestimate of the benefit of the budesonide/formoterol reliever therapy over budesonide/formoterol maintenance therapy.

Airway inflammation is variable in patients with asthma, suggesting the dose of anti-inflammatory treatment may need to vary in response. At times of relatively increased airway inflammation, a fixed dose ICS/LABA maintenance regime may provide an insufficient ICS dose and at times of relatively decreased airway inflammation, a fixed dose regime may provide an excessive ICS dose. The titration of ICS dose to severity of airway inflammation which can be achieved by an ICS/fast-onset LABA reliever regime, has the potential to reduce these periods or relative over and under treatment, which is reflected by the greater potency and efficacy respectively.

Severe exacerbations as defined by the ATS/ERS were the primary outcome variable used to assess potency and efficacy in this paper [8]. This is because severe exacerbations are generally regarded as a crucial asthma clinical outcome due to the resulting resource use and the strong association with an increased risk of mortality [19]. For secondary clinical outcome variables, measures of asthma control and lung function were utilised. For both potency and efficacy, there was a major difference between the regimens in terms of risk of severe exacerbations, whereas for asthma control and lung function, there was no major difference in efficacy. Differences in potency could not be assessed, however maintenance therapy was associated with a small but statistically significant improvement in FEV_1_ and ACQ score compared to budesonide/formoterol reliever therapy, both of which were below the minimal clinically important difference of 0.23 L [20] and 0.5 [21] respectively.

The estimates of ICS dose taken during the RCTs were calculated either from the dose counter on each inhaler, or self-completed diaries, which may be subject to inaccuracies of self-report. Perhaps of greater importance is the potential for enhanced treatment adherence to maintenance therapy in the context of a clinical trial, which would be expected to lead to an under-estimate of the comparative real world effect of budesonide/formoterol reliever therapy.

For the calculation of efficacy of the higher fixed dose maintenance budesonide/formoterol maintenance regimen, the budesonide dose was at the top of the dose-response curve [7]. This suggests that it was reasonable to use this randomised treatment as a comparator to assess the maximum obtainable benefit of the higher fixed dose maintenance budesonide/formoterol regimen.

A further study worthy of consideration was not eligible for inclusion in this review because it investigated ICS/SABA reliever monotherapy [22]. Combination beclomethasone diproprionate (BDP)/albuterol 250 μg/100 μg as required for relief of symptoms, was compared with twice daily BDP/albuterol 250 μg/100 μg with albuterol 100 μg as required for relief of symptoms [22]. The composite outcome of severe exacerbations comprising three variables, including the need for treatment with oral steroids, was reported. The number of severe exacerbations in the BDP/albuterol reliever and BDP/albuterol maintenance groups were 0/122 (0%) and 3/109 (2.8%) respectively. The zero cell count means that relative risk cannot be calculated, however the absolute risk difference (95% CI) was 2.8% (− 0.3 to 5.8%), *P* = 0.10 (Fishers exact test). The cumulative mean dose of BDP was 18.5 mg and 77.1 mg in the reliever and maintenance groups respectively, a dose ratio of 0.24. It therefore seems likely, even though a relative risk cannot be estimated, that the ICS/SABA reliever monotherapy regimen is considerably more potent than the maintenance ICS/SABA regimen. This is consistent with the potency estimates for ICS/LABA reliever monotherapy reported in our analysis.

## Conclusions

The limited evidence available indicates that budesonide/formoterol reliever therapy has greater potency and efficacy than regular maintenance budesonide/formoterol plus SABA reliever therapy in reducing the risk of severe exacerbations. Specifically, the results from our analysis indicate budesonide/formoterol reliever therapy can achieve the same effect as maintenance budesonide/formoterol at about one fifth of the dose, and achieve a greater maximum effect when added to maintenance budesonide/formoterol therapy compared with a higher maintenance fixed dose of budesonide/formoterol. Although based on a small number of studies, we consider this is an important concept which requires further investigation. We propose that knowledge of this difference in the dose-response relationship between fixed dose ICS/fast-onset LABA maintenance therapy and ICS/fast-onset LABA reliever therapy has the potential to guide evidence-based clinical practice.

## Data Availability

All data generated or analysed during this study are included in this published article.

## Abbreviations

ACQ: Asthma Control Questionnaire
ATS: American Thoracic Society
BDP: Beclomethasone diproprionate
ERS: European Respiratory Society
FEV_1_: forced expiratory volume in one second
ICS: inhaled corticosteroid
LABA: long acting beta agonist
RCT: randomised controlled trial
SABA: short acting beta agonist
